# Eclampsia

**Published:** 1898-12

**Authors:** M. L. Langford

**Affiliations:** Baileyville, Texas


### TEXAS MEDICAL JOURNAL.

ESTABLISHED JULY, 1885.

PUBLISHED MONTHLY.—SUBSCRIPTION $1.00 A YEAR.

VOL. XIV. AUSTIN, DECEMBER, 1898. No. 6.

### Original Contributions.

For Texas Medical Journal.

### Eclampsia.

BY M. L. LANGFORD, M. D., BAILEYVILLE, TEXAS.

Read before the Brazos Valley Medical Association, at its sixth semi-annual meeting at Rockdale, Texas, Nov. 15 and 16, 1898.

In the language of Boislinier, and I quote him verbatim, "There is no scene more intensely emotional and apalling than that presented by a woman suddenly stricken by an attack of eclampsia. Like a flash of lightning in a serene sky comes the convulsion, and as a violent hurricane it casts terror and dismay into the hearts of those witnessing it."

This affection, so rapid and so dangerous, has not failed to attract the attention of obstetricians in all ages. Its essential etiology is not settled beyond all controversy, nor is there perfect professional agreement in all things concerning the medical and obstetrical treatment. For these reasons, therefore, the subject is of great importance, and it demands careful consideration. Authorities differ greatly as to the per cent. of those attacked. Statistics show that as a rule it occurs about three or four times in every thousand patients. Many volumes have been written, and as many theories advanced, concerning the pathology and etiology. The first theory in the order of time is the albuminuria and pressure theory, advocated by Lever. This theory is based mainly upon the assumed compression of the renal arteries and veins by the gravid uterus. The arguments urged in its support are:

1st. The frequency of primipara, in whom the abdominal walls being firmer, press the uterus backward against the spine more forclbly.

2nd. The occurrence of edema and venectiosis of the lower extremities, pointing to pressure upon the vena cava.

3rd. The greater frequency when the uterus is of excessive size from twins or liquor amnii.

Against this general theory facts are in evidence.

1st. The pressure is inadequate, as the uterus, rising from the pelvis, diverges from the spinal column, leaving the kidneys and their blood-vessels protected in the receding lumbo-dorsal region; at most, the pressure is upon the common iliac veins and the lower part of the vena cava.

2nd. Albumen sometimes makes its appearance at the third or fourth month, before the uterus can possibly press upon the kidney or its vessels.

3rd. The albumen may disappear under treatment by purging or bleeding, although the uterus continues to grow; and again, albuminuria and convulsions occur in multipara, who escaped in their first pregnancy.

4th. The greater frequency in distension from twins may be more reasonably explained by the greater nervous and vascular tension induced by the double demand, and by the greater amount of excrematitious matter thrown into the circulation. These facts are not so conclusive against the theory of special pressure upon the ureters. Still, even here it may be objected that pressure upon the ureters will hardly account for eclampsia in the third and fourth months of gestation.

Another theory is, that eclampsia is caused by labor pains. This is occasionally true as regards the convulsion; it, however, fails to account for the albuminuria. When albuminuria already exists, then, as we have seen, uterine action may be the immediate cause of the convulsion. Often albuminuria and convulsion both occur without any sign of labor. Weyscheider collected 445 cases of eclampsia. Of these, 236 cases occurred during labor pains, 109 before, and 100 after labor. Of the 55 cases reported by Robt. Barnes, we find 18 cases in which the convulsions broke out without any antecedent sign of labor, labor being regarded by him as either an epiphenomenon caused by the convulsion, or induced by the physician.

Another theory has been advocated, and known as super albuminosis. The adherents of this theory believed that the eclampsia depended upon the excessive elimination of albumen with which the pregnant woman's blood was charged. To this quaint theory we reply by stating, if the proportion of albumin exceeds the normal wants of mother and fetus, the excess must accumulate in the blood and be eliminated by the kidneys, as is observed in animals fed exclusively on albumen, or in whose veins a solution of albumen is injected. The kidney, therefore, is not primarily diseased, but serves as a filter.

The theory of anemia, much vaunted by its advocates, is quite foreign to eclampsia. The convulsion of anemia from hemorrhage differs very materially from eclampsia. It is preceded by general tremor, a kind of universal shuddering; consciousness is not always abolished, and there is no trachelismus or congestion of the face. There is often emesis with rapid and imperceptible pulse, making a picture quite distinct from an eclamptic seizure.

The foregoing theories have all been exploded by diligent research of careful and painstaking obstetricians, and relegated one by one to the abyss of oblivion. While to-day we stand in the evening of the 19th century, with test tube in one hand and microscope in the other, catering to the whims of misdirected energies and courting the sacred precincts of confusion, I foster the thought that the period is not far distant when we will be able to classify, with precision, the microbe that produces the toxines that are now baffling our more modern investigators. These investigators have agreed upon a common factor, which they term toxæmia. With toxæmia as a factor, we are confronted with another series of theories to investigate and patly discard.

1st. The Uræmic Theory. Rostock, Gregory and Christison first noticed an excess of urea in the blood of eclamptic women, but it was left to Wilson, in 1833, to determine its morbid entity and declare the theory which contends that eclampsia is caused by an excess of urea in the blood, this excess acting as a poison to the nervous centers. This theory was successfully refuted by Claude Bernard, who proved the inoffensiveness of urea by injecting it into the veins without causing convulsions. Furthermore, we find, as additional proof, that the theory is erroneous, by making a comparison of the temperature of an eclamptic with that of an uræmic patient. In the latter, we find it becomes progressively lower, while in the eclamptic, it rises continually, and becomes very high.

We have another toxæmic theory known as ammonæmia, which eminated from the fertile brain of Frerich and is supported by very eminent authorities. Frerich contends that urea, under the influence of certain ferments in the blood, is transformed into ammonia carb., and that this compound produces the convulsion. Again we find Claude Bernard appear upon the scene, disprove this theory by proving that the blood, in sickness and in health, always contains more or less ammon. carb.; and, as further evidence, Monk, after tying the ureters of a dog, injected ammon. carb. without producing any untoward symptoms.

Schotten advocated the theory that excrementitious principles, derived from both mother and fetus, accompanying the urea, remain in the blood, produce its toxicity, resulting in the convulsion and coma. This seems a most rational supposition, and in connection with this theory we may, with due propriety, assert that a pregnant patient, whose system is thoroughly charged with miasmatic poison, is prone to suffer with eclampsia. My experience has been, that during periods that we were visited by the pernicious type of malaria, eclampsia was more prevalent. Several years ago our community suffered from a very malignant type of pernicious fever, and at the same time eclampsia was the universal rule, and not exceptional. Being a young practitioner at this time, I beheld these cases with evil forebodings, and wondered why my field of obstetric labor should be fraught with such a malady. I kept the history of these cases, noting the peculiar characteristics, treatment and results, for future reference, and for the purpose of elucidating the idea I wish to convey, will, with your permission, read them.

Case 1. Negress, age 33, mother of five children. Miscarried twice, last time three years prior to this confinement. Since then bore a healthy child and delivered at full time. Labor began at 10 a.m., or thereabout; had few scattering pains night before, at 12:30. Os uteri dilated size of silver twenty-five cent piece, and at this time was seized with first paroxysm, which was of comparatively short duration. Gave her hypodermic of one-half grain morphia and one-sixtieth grain atropia. At 1:30 ruptured membranes, and as liquor amnii gushed forth, another paroxysm came on, being a great deal harder and lasting much longer. I applied forceps, and delivered as quickly as possible, under chloroform. After removal of placenta, which was done by Crede method, patient suffered considerably from hemorrhage, for a few seconds. Patient, prior to confinement, had been troubled with hepatitis, or, more strictly speaking, passive hyperæmia. Liver was considerably swollen and very painful, stools were clay colored, and urine, at each examination, revealed bile pigment. Very little albumen noted at any time, though it was continuously present. Patient made rapid and uneventful recovery. Was the liver disorder that accompanied the gestation from its inception the cause of the eclampsia, and was the uterine hemorrhage the agent that cured the liver trouble?

CASE 2. Mrs. V., primipara, age 22, full habit. Was called at 2 p. m. Pains had been irregular for about twenty-four hours; intervals of two hours, etc. Face considerably swollen, feet and ankles so much so that her shoes were two sizes too small, and they had to split them. Skin very muddy, bowels constipated, urine heavily loaded with albumen and bile pigments. At 3:30, os uteri dilated about the size of fifty cents silver; convulsions occurred and kept up at intervals of twenty to thirty minutes. About 4 o'clock membranes ruptured, and labor hastened by instrumental delivery. Convulsions continued after child was delivered as before, intervals shorter, paroxysms longer; 6 p. m. convulsions occurring every ten minutes. Up to this hour, chloral had been given in ½-drachm doses at intervals of thirty minutes, mor. ½ gr. and atropia 1-120, at intervals of forty to sixty minutes. At 6 p. m., patient was bled to the amount of about twenty ounces. Paroxysm ceased for at least an hour, then recurred with greater force than ever. Patient was given elaterium, and at 7:30 bled from other arm. Convulsions were now, at second bleeding, occurring at intervals of five to ten minutes. Chloroform pushed to complete narcosis. Convulsions ceased for twenty minutes, and then returned with increased rigor, consciousness being fully lost, and continued every five to ten minutes up to death, at 10 p. m. Husband stated that his wife had been very bilious for past two or three months, and would frequently on arising in the morning, vomit pure bile, as he called it, and had to take something to get her bowels to move.

These two cases are sufficient, inasmuch as they represent the extremes.

If malaria will reduce a man in weight, impair his digestion, and, for the time, shatter his nervous system, destroy the oxygenating power of his blood by destroying red corpuscles, prevent the normal action of the liver, is it not probable that, when the atmospheric conditions are conducive to the generation of a malignant type of this special poison, there is some affinity between this poison and the malady under discussion, if occurring and ceasing? We have had no fever of a malarial type, except of the mildest form, since 1889, nor have I seen but one case of eclampsia since that date The treatment is about what was stated in the cases cited.

